# Maternally Orphaned Children and Intergenerational Concerns Associated With Breast Cancer Deaths Among Women in Sub-Saharan Africa

**DOI:** 10.1001/jamaoncol.2020.6583

**Published:** 2020-12-23

**Authors:** Moses Galukande, Joachim Schüz, Benjamin O. Anderson, Annelle Zietsman, Charles Adisa, Angelica Anele, Groesbeck Parham, Leeya F. Pinder, Songiso Mutumba, Dorothy Lombe, Anna Cabanes, Milena Foerster, Isabel dos-Santos-Silva, Valerie McCormack

**Affiliations:** 1 Department of Surgery, College of Health Sciences, Makerere University, Kampala, Uganda; 2 Branch of Environment and Lifestyle Epidemiology, International Agency for Research on Cancer, Lyon, France; 3 Fred Hutchinson Cancer Research Center and University of Washington, Seattle; 4 Windhoek Central Hospital, Namibia; 5 Department of Surgery, Abia State University Teaching Hospital Nigeria, Aba, Abia, Nigeria; 6 Breast Oncology Unit, Federal Medical Centre Owerri, Nigeria; 7 UNC Department of Obstetrics and Gynecology, University of Zambia, Lusaka, Zambia; 8 Department of Obstetrics and Gynecology, University of Washington, Seattle; 9 Outpatient Department, Matero Level One Hospital, Lusaka, Zambia; 10 Department of Oncology, Cancer Diseases Hospital, Lusaka, Zambia; 11 Susan G. Komen, Dallas, Texas; 12 Branch of Environment and Lifestyle Epidemiology, International Agency for Research on Cancer, Lyon, France; 13 Department of Non-Communicable Diseases Epidemiology, London School of Hygiene and Tropical Medicine, London, United Kingdom

## Abstract

**Importance:**

Low breast cancer survival in sub-Saharan Africa’s young population increases the likelihood that breast cancer deaths result in maternal orphans, ie, children (<18 years) losing their mother.

**Objective:**

To estimate the number of maternal orphans and their ages for every 100 breast cancer deaths in sub-Saharan African settings during 2014-2019 and to describe family concerns about the orphaned children.

**Design, Setting, and Participants:**

Deaths occurring between September 1, 2014, and July 1, 2019, in the African Breast Cancer–Disparities in Outcomes (ABC-DO) were examined in a cohort of women diagnosed with breast cancer during 2014-2017 at major cancer treatment hospitals in Namibia, Nigeria, Uganda, and Zambia. The cohort was actively followed up for vital status via a trimonthly mobile phone call to each woman or her next of kin (typically a partner, husband, or child).

**Main Outcomes and Measures:**

The number (Poisson counts) and ages of new orphans at the time of maternal death.

**Results:**

This cohort study found that a total of 795 deaths resulted in 964 new maternal orphans, with deaths occurring in women younger than 50 years accounting for 85% of the orphans. For every 100 deaths in women younger than 50 years, there were 210 new orphans (95% CI, 196-225) overall, with country-specific estimates of 189 in Nigerian, 180 in Namibian, 222 in Ugandan, and 247 in Zambian Black women. For every 100 deaths of the women at any age, there were 121 maternal orphans, 17% of whom were younger than 5 years, 32% aged 5 to 9 years, and 51% aged 10 to 17 years at the time of maternal death. In follow-up interviews, families’ concerns for children’s education and childcare were reported to be exacerbated by the financial expenses associated with cancer treatment.

**Conclusions and Relevance:**

This study provides evidence that the number of maternal orphans due to breast cancer exceeds the number of breast cancer deaths among women in sub-Saharan Africa. The intergenerational consequences associated with cancer deaths in sub-Saharan Africa appear to be large and support the need for continued action to improve survival.

## Key Points

**Question:**

How many maternal orphans resulted from every 100 breast cancer deaths in sub-Saharan African settings in 2014-2019?

**Findings:**

In the African Breast Cancer–Disparities in Outcomes cohort study of women with breast cancer, there were 964 children (<18 years) remaining at the time of maternal death and 795 deaths of women in Namibia, Nigeria, Uganda, and Zambia. Half of these maternal deaths occurred in women younger than 50 years and, for every 100 breast cancer deaths in this age group, 210 children became maternal orphans.

**Meaning:**

The results of this study quantify the intergenerational consequences associated with cancer death among children in Sub-Saharan Africa whose mothers die from breast cancer.

## Teaser

This cohort study estimates the number of maternal orphans in 4 sub-Saharan Africa countries when women die of breast cancer.

## Introduction

In sub-Saharan Africa, where cancer survival is relatively low,^1^ families affected by cancer are more likely to experience a death than in other world regions. With sub-Saharan Africa’s young population and high fertility rates,^2^ the deaths of middle-aged mothers and fathers result in maternal and paternal orphans (children <18 years at parental death). Breast cancer accounts for 24% of cancer deaths in sub-Saharan African women.^3^ Although half (48%) of these deaths occur in women younger than 50 years (eFigure in the Supplement), we are not aware of quantifications of the resulting maternal orphans. In the present study, we examined these numbers in the African Breast Cancer–Disparities in Outcomes (ABC-DO) cohort^4,5^ and highlight the outcomes of the deaths in families.

## Methods

### Study Design and Participants

The ABC-DO study evaluates a hospital-based breast cancer cohort in sub-Saharan Africa to estimate overall survival and its determinants; the protocol has been published,^4^ and ethics approval was obtained from all national or institutional review boards (eAppendix in the Supplement). This study followed the Strengthening the Reporting of Observational Studies in Epidemiology (STROBE) reporting guideline. Participating hospitals included in this analysis were the only public cancer treatment hospitals in Namibia, Uganda, and Zambia, located in their capital cities, and 2 hospitals and a clinic in Nigeria’s Imo and Abia states. All women aged 18 years or older with incident histologically or clinically diagnosed breast cancer between September 1, 2014, and December 31, 2017, were invited to participate, of whom 99% provided written informed consent (stating that published information would not identify women). Participants did not receive financial compensation. Women were included regardless of cancer stage, intention to undergo treatment, or residential location. The baseline interview included information on the woman’s age, number of children and the woman’s age at the first and last live births, self-reported HIV status, and because these factors differ by race, self-assigned ethnicity in Namibia where Black ethnicities were combined and White and mixed-race women were grouped into a non-Black women category.

The cohort was actively followed up for vital status via a trimonthly mobile phone call to each woman or her next of kin (typically a partner, husband, or child), an approach that minimized losses to follow-up.^6^ When the next of kin reported a death (91% of deaths), they were asked how the death affected the family, and free-text responses were classified into emotional consequences, financial concerns, and other concerns related to children. In the present analysis, we estimated the number of maternal orphans associated with deaths occurring until July 1, 2019 (median, 3.5 years [interquartile range, 2.9-4.2 years] after recruitment had women survived).

### Statistical Analysis

To estimate the number of maternal orphans, we first calculated the ages of the woman’s children from the first and last live births at the date of her death (eMethods and eTable 1in the Supplement). The ages of children from any middle live births were assumed with equal spacing between the first and last child. Children’s ages were used to calculate the number of each woman’s maternal orphans at her death. Thereafter, because children of all live births were not necessarily still alive, if a woman indicated at her most recent contact that fewer children lived with her, the number of maternal orphans was reduced to this lower number. Determinants of the number of orphans were modeled using Poisson regression models, and Wald tests were used to obtain *P* values for linear trends in education and for differences by residence and HIV status. Significance was determined using 2-sided tests with a 5% threshold. Statistical analysis was conducted using Stata, version 15 (StataCorp LLC). Further statistical details are provided in the eMethods in the Supplement.

## Results

Of the 1541 cohort members, mean (SD) age at diagnosis was 50.4 (14.3) years, and 149 women (10%) were HIV-positive. Breast cancer diagnoses in women with data available were stages I/II in 523 (36%), stage III in 688 (48%), and stage IV in 225 (16%) women. Three-year survival was 50%, and published determinants of survival include late stage, treatment gaps, young age (<30 years), low educational level, positive HIV status, and triple-negative tumors.^5^ In total, 795 women had died by July 1, 2019: 256 Nigerian women, 249 Ugandan women, 185 Namibian Black women, 86 Zambian women, and 19 Namibian non-Black women (eTable 2 in the Supplement). A total of 390 of the 795 deaths (49%) occurred in women younger than 50 years. A total of 711 women (89%) who died had been parous (country range, 81%-94%) (eTable 2 in the Supplement). Among those older than 40 years when they died, mean (SD) age at the last birth ranged from 34 (5.9) years in the more recent to 39 (7.1) years in the earlier birth cohort, ie, giving rise to offspring who may still be minors if the mother died in or before her 50s. The median number of live births was 3 to 4 in Namibia and Nigeria and between 4 and 7 in Uganda and Zambia, but was lower in women who died when they were younger than 40 years owing to their shortened reproductive life span. Namibian non-Black women had lower parity (median, 2 live births) (eTable 3 in the Supplement).

These 795 deaths resulted in 964 new maternal orphans, ie, 121 maternal orphans per 100 deaths, ranging from 96 (95% CI, 83-111) in Namibian Black women to 145 (95% CI, 131-161) in Ugandan women (Table 1). Deaths in women younger than 50 years resulted in 819 maternal orphans (85%). At these ages there were 210 (95% CI, 196-225) orphans per 100 breast cancer deaths overall, with the highest estimates in Uganda (222) and Zambia (247), followed by 189 in Nigeria and 180 in Namibia (Figure). Most (494) orphans (51%) were older than 10 years at their mothers’ death, 311 (32%) were aged 5 to 9 years, and 160 (17%) were younger than 5 years. Deaths of women from lower educational level groups and from rural areas (186; 95% CI, 170-203) were associated with more orphans, whereas, owing to their lower parity, deaths of HIV-positive women (122; 95% CI, 99-150) resulted in 33% fewer orphans than of HIV-negative women (175; 95% CI, 163-187) (Table 2).

**Table 1.** Deaths and Resulting Maternal Orphans Among Women in the ABC-DO Study, 2014-2019, by Country and Age at Death^a^

Country	Age at breast cancer death, y				
	<40	40-49	50-59	≥60	All ages
**Deaths, No. (%)**					
All	169 (21)	221 (28)	187 (24)	218 (27)	795 (100)
Namibia (Black)	27 (15)	44 (24)	42 (23)	72 (39)	185 (100)
Namibia (non-Black)	1 (5)	4 (21)	5 (26)	9 (47)	19 (100)^a^
Nigeria	61 (24)	71 (28)	62 (24)	62 (24)	256 (100)
Uganda	61 (25)	83 (33)	61 (25)	44 (18)	249 (100)
Zambia	19 (22)	19 (22)	17 (20)	31 (36)	86 (100)
**Women who died leaving minor children, No. (%)**					
All	128 (74)	174 (79)	77 (41)	4 (2)	383 (48)
Namibia (Black)	20 (74)	38 (86)	16 (38)	1 (1)	75 (41)
Nigeria	37 (61)	52 (73)	23 (37)	2 (3)	114 (45)
Uganda	54 (89)	65 (78)	28 (46)	0 (0)	147 (59)
Zambia	16 (84)	18 (95)	9 (53)	1 (3)	44 (48)
**Maternal orphans, No. (%)**					
All	354 (37)	465 (48)	141 (15)	4 (0.4)	964 (100)
Namibia (Black)	56 (31)	91 (51)	30 (17)	1 (0.6)	178 (100)
Nigeria	102 (34)	148 (49)	47 (16)	2 (0.7)	299 (100)
Uganda	150 (41)	169 (47)	43 (12)	0 (0)	362 (100)
Zambia	43 (37)	51 (44)	20 (17)	1 (0.9)	115 (100)
**Maternal orphans <18 y per 100 breast cancer deaths, No. (95% CI)**					
All	209 (188-232)	210 (192-230)	75 (63-89)	2 (0-5)	121 (114-129)
Namibia (Black)	207 (157-269)	207 (167-254)	71 (48-102)	1 (0-8)	96 (83-111)
Nigeria	167 (136-203)	208 (176-245)	76 (56-101)	3 (0-12)	117 (104-131)
Uganda	246 (208-289)	204 (174-237)	70 (51-95)	0 (0-8)	145 (131-161)
Zambia	226 (164-305)	268 (200-353)	118 (72-182)	3 (0-18)	134 (110-161)
**No. of maternal orphans <10 y per 100 breast cancer deaths (95% CI)**					
All	148 (130-167)	92 (80-105)	10 (6-16)	0 (0-2)	59 (54-65)
Namibia (Black)	152 (109-206)	82 (57-113)	14 (5-31)	0 (0-5)	45 (36-56)
Nigeria	134 (107-167)	99 (77-125)	10 (4-21)	0 (0-6)	62 (52-72)
Uganda	161 (130-196)	86 (67-108)	3 (0-12)	0 (0-8)	69 (59-80)
Zambia	137 (89-201)	121 (77-182)	29 (10-69)	0 (0-12)	63 (47-82)
**No. of maternal orphans <5 y per 100 breast cancer deaths (95% CI)**					
All	67 (56-81)	20 (15-27)	1 (0-4)	0 (0-2)	20 (17-24)
Namibia (Black)	67 (40-105)	16 (6-33)	2 (0-13)	0 (0-5)	14 (9-21)
Nigeria	69 (50-93)	21 (12-35)	0 (0-6)	0 (0-6)	22 (17-29)
Uganda	66 (47-89)	19 (11-31)	0 (0-6)	0 (0-8)	22 (17-29)
Zambia	63 (33-110)	32 (12-69)	6 (0-33)	0 (0-12)	22 (13-35)

Abbreviation: ABC-DO, African Breast Cancer–Disparities in Outcomes.

^a^ There were 10 maternal orphans associated with the 19 breast cancer deaths in non-Black Namibian women. These are not shown for site-specific strata in the remainder of the table owing to small numbers and identifiability, but were included in the estimates of number of orphans per 100 deaths for all sites combined. Thus, the numerators/denominators differ for some factors.

**Figure.**
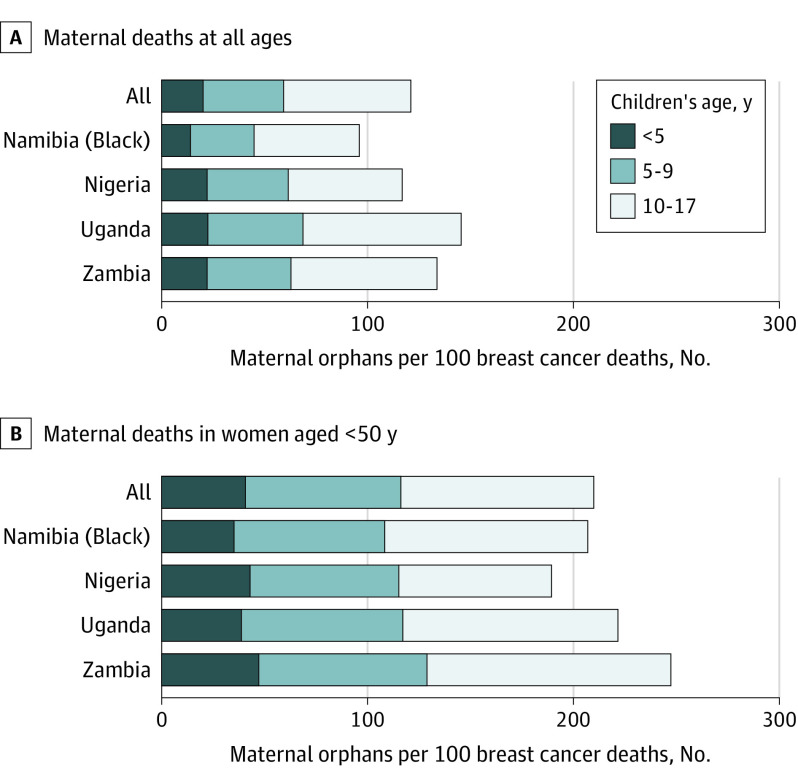
Country-Specific and Overall Number of Maternal Orphans Resulting From Every 100 Breast Cancer Deaths

**Table 2.** Mean Number of New Maternal Orphans Resulting From 100 Breast Cancer Deaths in Women Younger Than 60 Years

Variable	New maternal orphans per 100 breast cancer deaths in women aged <60 y, No.					Ratio	
	Namibia Black	Nigeria	Uganda	Zambia	All	Adjusted^a^	Adjusted^b^
No. deaths	113	194	205	55	567		
No. of orphans	157 (134-181)	153 (136-172)	177 (159-196)	207 (171-249)	168 (157-179)	NA	NA
Education							
None/primary	175 (141-213)	126 (92-170)	179 (155-206)	236 (180-304)	176 (159-195)	1.42 (1.16-1.75)	1.30 (1.05-1.61)
Secondary	145 (112-183)	173 (146-203)	181 (153-213)	187 (135-252)	171 (155-189)	1.19 (0.98-1.44)	1.16 (0.96-1.41)
Technical/university	118 (62-202)	143 (117-172)	138 (86-208)	171 (89-299)	141 (120-165)	1 [Reference]	1 [Reference]
*P* value for trend	.11	.92	.47	.19	.13		
Residential area							
Urban	146 (119-178)	140 (121-162)	127 (97-163)	219 (174-273)	149 (136-164)	1 [Reference]	1 [Reference]
Rural	173 (136-216)	182 (149-219)	192 (171-215)	184 (128-256)	186 (170-203)	1.32 (1.15-1.52)	1.25 (1.08-1.45)
*P* value	.24	.08	.004	.73	.002		
HIV							
Negative/unknown	169 (143-198)	154 (137-173)	189 (169-210)	224 (179-277)	175 (163-187)	1 [Reference]	1 [Reference]
Positive	113 (74-164)	117 (47-240)	1037 (70-148)	116 (114-245)	122 (99-150)	0.65 (0.53-0.81)	0.67 (0.54-0.84)
*P* value	.05	.47	.002	.21	.003		

Abbreviation: NA, not applicable.

^a^ Adjusted for country and age at death.

^b^ Adjusted for country, age at death, educational level, HIV status, and rural residence.

The next of kin’s reports of how the woman’s death affected the family highlighted emotional and financial results (eTable 4 in the Supplement). Concerns for children and their education and care were also common. Financial worries were exacerbated by a lack of assets to pay for cancer treatment. Grandparents were mentioned as stepping in to provide childcare. Typically, hardships often clustered, as illustrated in this quotation: “She left a younger child of 2 years with no help yet most of the land was sold in order to buy the prescribed drugs for her treatment.”

## Discussion

More than half a million breast cancer deaths are projected to occur in sub-Saharan African during 2020-2029.^7^ This analysis suggests that at least as many new maternal orphans will result from these deaths, ie, highlighting the intergenerational effects of cancer unique to young populations with low survival. The number of maternal orphans was associated with maternal deaths in women younger than 50 years, and owing to higher fertility levels, particularly affected HIV-negative women and women in rural communities. Lower socioeconomic groups, which have lower breast cancer survival,^5^ result in more maternal orphans, thus propagating a cycle of disadvantage.

### Limitations

Our estimates of maternal orphans are based on a large number of patients attending major cancer treatment centers in 4 countries and, to our knowledge, are the only available estimates at present. However, because ABC-DO was not designed a priori to address the number of maternal orphans, future studies of this issue can be improved by including population-based cases and noncapital settings, by directly ascertaining the numbers and ages of children and the familial care situation. Although ABC-DO deaths occurred within 4 years of diagnosis, standardization to the Globocan 2018 age at death distribution did not materially affect the results (eTable 5 in the Supplement).The ABC-DO findings indicated a high percentage of unmarried women; we did not have information on other partner/adult support and caregivers. Studies with prospective follow-up of children’s development and care should also be undertaken.

## Conclusions

To minimize the intergenerational effects of breast cancer deaths in sub-Saharan Africa, preventing breast cancer deaths through early diagnosis and improved timely treatment should form an essential part of every cancer control plan.^8,9,10^ In parallel, support mechanisms are needed for affected families because maternal deaths impact the children’s education,^11^ development, nutrition, and mortality,^12^ as described in a joint report by the United Nations Programme on HIV/AIDS, the United Nations Children’s Fund, and the US Agency for International Development.^13^ Because women are the cornerstone of many African families, there is often great upheaval after their death. If the father remarries, children usually remain with their father; otherwise, they may be cared for by close relatives or in care homes. In sub-Saharan Africa, lessons can be borrowed from those gained during the HIV epidemic, such as leaving memories (eg, photographs of the parents) for children and of models of care.^14^ Cancer support associations also play important roles in relieving pressures on families, and some cancer associations support the costs of education for orphans.^15^

## Notes

Supplement. eAppendix. Ethics Approvals for ABC-DO **eMethods.** Analysis of the Number of Maternal Orphans at the Time of Maternal Death **eTable 1.** Methods to Estimate the Number of Maternal Orphans at the Time of Maternal Death **eTable 2.** Demographic and Family Characteristics of Women Who Died, ABC-DO Breast Cancer Cohort, by Study Site, Race in Namibia, and Age at Death **eTable 3.** Reproductive Factors and HIV Prevalence, by Age at Death for Each Country-Race Group **eTable 4.** Qualitative Information on the Impact of the Breast Cancer Death on the Family **eTable 5.** Comparison of the Age-at-Death Distribution in ABC-DO With Globocan 2018 Estimates for the Corresponding Country and Estimates of Maternal Orphans Adjusted to the Globocan Age-at-Death Distributions **eFigure.** Distribution of Age at Breast Cancer Death in Each WHO Region, noting the Percentage of Breast Cancer Deaths Occurring <50 Years
