# Supplementary material for: Maternally Orphaned Children and Intergenerational Concerns Associated With Breast Cancer Deaths Among Women in Sub-Saharan Africa
Source: JAMA Oncol. 2020 Dec 23;7(2):1–5. doi: 10.1001/jamaoncol.2020.6583 (PMC7758819; doi:10.1001/jamaoncol.2020.6583)
Supplement: Supplement. — eAppendix. Ethics Approvals for ABC-DO eMethods. Analysis of the Number of Maternal Orphans at the Time of Maternal Death eTable 1. Methods to Estimate the Number of Maternal Orphans at the Time of Maternal Death eTable 2. Demographic and Family Characteristics of Women Who Died, ABC-DO Breast Cancer Cohort, by Study Site, Race in Namibia, and Age at Death eTable 3. Reproductive Factors and HIV Prevalence, by Age at Death for Each Country-Race Group eTable 4. Qualitative Information on the Impact of the Breast Cancer Death on the Family eTable 5. Comparison of the Age-at-Death Distribution in ABC-DO With Globocan 2018 Estimates for the Corresponding Country and Estimates of Maternal Orphans Adjusted to the Globocan Age-at-Death Distributions eFigure. Distribution of Age at Breast Cancer Death in Each WHO Region, noting the Percentage of Breast Cancer Deaths Occurring <50 Years [file jamaoncol-e206583-s001.pdf]

Supplementary Online Content

Galukande M, Schüz J, Anderson BO, et al. Maternally orphaned children and intergenerational concerns associated with breast cancer deaths among women in sub-Saharan Africa. *JAMA Oncol*. Published online December 23, 2020. doi:10.1001/jamaoncol.2020.6583

eAppendix. Ethics Approvals for ABC-DO

eMethods. Analysis of the Number of Maternal Orphans at the Time of Maternal Death

eTable 1. Methods to Estimate the Number of Maternal Orphans at the Time of Maternal Death

eTable 2. Demographic and Family Characteristics of Women Who Died, ABC-DO Breast Cancer Cohort, by Study Site, Race in Namibia, and Age at Death

eTable 3. Reproductive Factors and HIV Prevalence, by Age at Death for Each Country-Race Group

eTable 4. Qualitative Information on the Impact of the Breast Cancer Death on the Family

eTable 5. Comparison of the Age-at-Death Distribution in ABC-DO With Globocan 2018 Estimates for the Corresponding Country and Estimates of Maternal Orphans Adjusted to the Globocan Age-at-Death Distributions

eFigure. Distribution of Age at Breast Cancer Death in Each WHO Region, noting the Percentage of Breast Cancer Deaths Occurring <50 Years

This supplementary material has been provided by the authors to give readers additional information about their work.

eAppendix. Ethics approvals for ABC-DO

International Agency for Research on Cancer: IEC13-19, IEC15-18
London School of Hygiene and Tropical Medicine, UK: 6459
Nigeria: Federal Medical Centre Owerri
Nigeria: Abia State University Teaching Hospital
Zambia: University of Zambia Biomedical Research Ethics Committee (004-08-15)
Uganda: Uganda National Council for Science and Technology (HS 1588)
Namibia: Ministry of Health and Social Services of Namibia (17/3/3)

eMethods. Analysis of the Number of Maternal Orphans at the Time of Maternal Death

The number of maternal orphans for each woman was first calculated based on the ages of each of her live births at the time of her death (Approach 1 in the eMethods eTable 1 below). For 50% of women, all live births would have been adults by the time of their mother's death, thus no maternal orphans were associated with these deaths. For the remaining 50% of women, at least one live birth would have been a minor. This approach led to an estimate of 1035 maternal orphans associated with the 795 deaths. In Approach 2, a more conservative estimate of the number of maternal orphans was made, by reducing the estimate of maternal orphans made in Approach 1 if the woman had indicated, at the baseline interview or at her most recent follow-up interview prior to death, that less children were living with her. This approach reduced the maternal orphans for 44 women (5.5%) by 71 orphans, i.e. a reduction of 7% in the total number of orphans from 1035 to 964. Where the number of orphans was reduced, a woman's youngest children were assumed to be still with her.

Statistical analysis: Poisson regression models were used to estimate 95% confidence intervals for the number of maternal orphans and to examine correlates of the number of orphans. These estimates were made overall, then by country (and race in Namibia) and additionally stratified by age at maternal death (<40, 40-49, 50-59, and 60+ years). If there were fewer than 10 deaths in a specific stratum, which applied to Namibian non-black women who were a small group with high survival, the number of orphans was not provided to preserve confidentiality. The potential of overestimating orphans due to the short follow-up period was investigated by standardizing to the Globocan 2018 age distribution of cancer deaths in each country[4]. These Globocan-standardized estimates were very similar to crude estimates overall (eTable 5). Globocan-standardized estimates of maternal orphans for Uganda and Zambia were 13% lower than those corresponding values obtained in the ABC-DO cohort, due to the latter having slightly younger age distributions than Globocan. However, the estimates of maternal orphans associated with deaths under age 50 were unaltered.

eTable 1. Methods to estimate the number of maternal orphans at the time of maternal death

Category	Approach 1 (live birth information only)			Approach 2 (live births updated with children living at home)		
	Details	No. women (%)	No. maternal orphans	Details	No. women (%)	No. maternal orphans
No live births	No maternal orphans	84 (10.6)	0	No maternal orphans	84 (10.6)	0
All live births would be >18 y at maternal death	No maternal orphans	317 (39.9)	0	No maternal orphans	317 (39.9)	0
At least one live birth would be < 18 y	Calculate no. maternal orphans based on ages of each live birth, of which:			Calculate no. maternal orphans based on ages of each live birth and reduce this number if:<br>(A) at the most recent follow-up contact[a], the woman indicated she was living with less children, resulting in:		
	All live births <18 at maternal death	168 (21.1)	470	No change	265 (33.3)	651
				Reduction in maternal orphans by 1	19 (2.4)	42
				Reduction in maternal orphans by 2	9 (1.1)	24
				Reduction in maternal orphans by 3	4 (0.5)	5
	Some but not all live births <18 at maternal death	226 (28.4)	565	(B) (for women without an interim follow-up contact) at the baseline interview[b], the woman indicated she was living with less children, which resulted in:		
				No change	85 (10.7)	225
				Reduction in maternal orphans by 1	4 (0.5)	3
				Reduction in maternal orphans by 2	6 (0.8)	8
				Reduction in maternal orphans by 3	2 (0.3)	5
Total		795 (100)	1035		795 (100)	964
Orphans per 100 breast cancer deaths (95% CI)	130 (122, 138)			121 (114, 129)		

[a] for these 97 women, median (IQR) time from baseline interview to death was 4.3 months (1.1, 4.0). [b] for these 297 women, median (IQR) time from baseline interview to death was 17.6 months (10.1, 26.3).

eTable 2: Demographic and family characteristics of women who died, ABC-DO breast cancer cohort, by study site, race in Namibia, and age at death

N (col %)	Category	Namibia black	Namibia non black	Nigeria	Uganda	Zambia	All
Number of women who died	-	185	19	256	249	86	795
**Baseline information at the time of diagnosis for women who died during 01Sept 2014 to 31 Jul2019**							
Age at cancer diagnosis (y)	Mean (SD)	54.7 (16.2)	55.9 (14.1)	48.9 (12.7)	47.5 (12.7)	52.9 (16.3)	50.4 (14.3)
Educational level	None	46 (25)	0	20 (8)	42 (17)	15 (17)	123 (15)
	Primary	68 (37)	6 (32)	60 (23)	106 (43)	36 (42)	276 (35)
	Secondary	58 (31)	9 (47)	93 (36)	83 (33)	25 (29)	268 (34)
	Tertiary	13 (7)	4 (21)	83 (32)	18 (7)	10 (12)	128 (16)
Residential location	Urban	97 (52)	15 (79)	164 (64)	53 (21)	48 (56)	377 (47)
	Rural	88 (48)	4 (21)	92 (36)	196 (79)	38 (44)	418 (53)
Children	At least one child < 18 y	82 (44)	4 (21)	122 (48)	160 (64)	45 (52)	413 (52)
	All children aged 18+ y	90 (49)	13 (68)	85 (33)	74 (30)	36 (42)	298 (37)
	Never had a live birth	13 (7)	2 (11)	49 (19)	15 (6)	5 (6)	84 (11)
Pregnant at diagnosis	Yes N (%)	2 (1.1)	1 (5.3)	7 (2.7)	4 (1.6)	0 (0)	14 (1.8)
Marital status at diagnosis	Married/cohabiting	43 (23)	11 (58)	164 (64)	117 (46)	48 (56)	382 (48)
	Single	84 (45)	1 (4)	32 (13)	20 (8)	7 (8)	144 (18)
	Divorced	15 (8)	4 (21)	6 (2)	46 (18)	5 (6)	76 (10)
	Widowed	42 (23)	3 (16)	53 (21)	52 (21)	25 (29)	175 (22)
	Other	1 (<1)	0 (0)	1 (<1)	15 (6)	1 (1)	18 (2)
**Mortality information**							
Age at death (years)	Mean (SD)	56.2 (16)	57.7 (14)	50.1 (13)	48.9 (13)	53.8 (16)	51.7 (14)
Place of death (2 missing)	Home	42 (23)	6 (32)	203 (79)	163 (66)	30 (35)	444 (56)
	Hospital/hospice	140 (76)	12 (63)	51 (20)	65 (26)	55 (64)	323 (41)
	Other	2 (1)	1 (5)	2 (1)	20 (8)	1 (1)	26 (3)
HIV, deaths < 50 years	HIV positive	16 (23)	0	5 (4)	20 (14)	10 (26)	51 (13)
HIV, deaths ≥ 50 years	HIV positive	12 (11)	1 (7)	1 (1)	10 (10)	8 (17)	32 (8)

eTable 3: Reproductive factors and HIV prevalence, by age at death for each country-race group

Site-race group	Age at death (y)	No. deaths	Age of women at her first birth		Age of woman at her last birth		Live births (percentiles)			HIV prevalence
		N	Mean	SD	Mean	SD	50th	25th	75th	proportion
Namibia, black	15-39	27	21.6	4.2	28.1	4.0	2	1	3	0.15
Namibia, black	40-49	44	21.8	3.9	33.9	5.9	3	2	4.5	0.27
Namibia, black	50-59	42	21.3	4.1	35.3	6.8	4	2	5	0.19
Namibia, black	60+	72	22.3	4.8	36.4	5.8	6	4	8	0.06
Namibia, non-black*	15-39	5	22.8	3.3	29.0	3.7	2	2	2	0
Namibia, non-black*	40-49	23	24.4	5.6	32.1	5.4	2	2	3	0.04
Namibia, non-black*	50-59	29	22.4	4.2	30.7	5.7	2	2	3	0.07
Namibia, non-black*	60+	44	23.2	3.8	30.9	5.0	3	2	4	0
Nigeria	15-39	61	25.8	4.7	30.8	4.6	1	0	3	0.03
Nigeria	40-49	71	26.8	6.1	35.2	4.5	3	1	5	0.04
Nigeria	50-59	62	25.6	6.3	35.1	5.8	4	1	5	0.02
Nigeria	60+	62	24.4	4.6	37.1	5.4	6	4	8	0.00
Uganda	15-39	61	20.5	3.6	28.6	4.0	3	2	4	0.10
Uganda	40-49	83	20.4	4.6	33.3	6.6	4	2	6	0.17
Uganda	50-59	61	18.7	2.9	34.5	7.8	6	4	7	0.15
Uganda	60+	44	19.0	4.5	38.4	6.8	7.5	4	10	0.02
Zambia	15-39	19	20.9	3.8	29.7	4.3	3	1	4	0.21
Zambia	40-49	19	20.8	6.0	37.7	3.7	5	3	6	0.32
Zambia	50-59	17	20.6	6.7	38.8	7.1	7	4	8	0.41
Zambia	60+	31	19.2	2.7	39.2	7.1	8	5	9	0.03

In non-black Namibian women, statistics are provided for all 101 women with breast cancer in the cohort, as there were too few (19) deaths, where age at diagnosis was used for women still alive on the closing date.

eTable 4: Qualitative information on the impact of the breast cancer death on the family

Impact (N=frequency*)	Examples
Emotional<br>(N=167)	Emotional, sad, anguish, pain, distress, devastation, sadness, distraught, depression, shock, unexpected death, anger
Concerns about loss of mother figure, her support, child's education and care<br><br>(N=33)	• "She was the only one who was taking care of her children, so her children may not continue with the education she had wished for them."<br>• "She was supporting her family, paying school fees for her children so now they may not get the education she wanted."<br>• "Younger children left with old parents, grandmother raising 7 kids";<br>• "6 young children in primary school"<br>• "children were left alone whereby they had to be taken by relatives."<br>• "she had a family of nine children who were left helpless because she was a single mother"<br>• "she was a pillar in the family"<br>• "no mother figure";<br>• "Husband was very sad and worried because he was left with 6 small children and he didn't know how he would bring them up without the mother."
Financial<br><br>(N=29)	• "family missed her much because she was the source of income"<br>• "She left a younger child of 2 years with no help yet most of the land was sold in order to buy the prescribed drugs for her treatment."<br>• Burial costs, demands from family: "financial involvement towards her burial and burial rite and demands from her extended family and in-laws"<br>• Physical challenges, removing corpse<br>• "The family was upset by her death since they tried their best to save her life whereby they used a lot of money to the extent of selling most of their land but she died."<br>• "travelled a long distance to seek treatment and all was too late"

* multiple categories can apply to a single family.

eTable 5: Comparison of the age-at-death distribution in ABC-DO with Globocan 2018 estimates for the corresponding country and estimates of maternal orphans adjusted to the Globocan age-at-death distributions

		All			Namibia (black)			Nigeria			Uganda			Zambia		
Age at death (y)		ABCDO-all 795 deaths	Globocan SSA	Difference	ABC-DO	Globocan SSA	Difference	ABC-DO	Globocan SSA	Difference	ABC-DO	Globocan SSA	Difference	ABC-DO	Globocan SSA	Difference
% distribution																
<15		0.0	0.0	0.0	0.0	0.0	0.0	0.0	0.0	0.0	0.0	0.0	0.0	0.0	0.0	0.0
15-29		3.9	2.8	1.1	2.0	0.8	1.1	3.1	3.4	-0.3	5.6	1.0	4.6	5.8	0.8	5.0
30-39		17.4	19.2	-1.8	11.8	13.1	-1.4	20.7	21.8	-1.1	18.9	17.5	1.4	16.3	12.5	3.8
40-49		27.8	26.7	1.1	23.5	23.0	0.6	27.7	30.6	-2.8	33.3	29.5	3.9	22.1	21.6	0.5
50-59		23.5	23.0	0.5	23.0	22.1	0.9	24.2	24.9	-0.7	24.5	26.7	-2.2	19.8	21.9	-2.1
60-69		15.6	15.9	-0.3	19.6	18.0	1.6	18.4	14.3	4.1	10.0	18.1	-8.1	14.0	22.2	-8.2
70-79		7.7	8.6	-0.9	10.3	13.1	-2.8	3.5	4.7	-1.1	6.4	6.6	-0.2	17.4	15.2	2.2
80+		4.2	3.9	0.3	9.8	9.8	0.0	2.3	0.3	2.0	1.2	0.7	0.5	4.7	5.8	-1.2
**Total**		**100.0**	**100.0**		**100.0**	**100.0**		**100.0**	**100.0**		**100.0**	**100.0**		**100.0**	**100.0**	
Mean age at death		51.9	52.1	-0.2	56.7	57.3	-0.6	50.3	48.9	1.4	48.7	51.6	-2.8	54.0	57.1	-3.2
% of deaths < 50 y		49.1	48.7	0.4	37.3	36.9	0.4	51.6	55.8	-4.2	57.8	48.0	9.9	44.2	34.9	9.3
		BC deaths			BC deaths			BC deaths			BC deaths			BC deaths		
No. maternal orphans per 100 breast cancer deaths		All ages	< 50 y		All ages	< 50 y		All ages	< 50 y		All ages	< 50 y		All ages	< 50 y	
Crude estimate		121	210		94	207		117	189		145	222		134	247	
Weighted to Globocan		120	210		92	207		125	189		124	220		115	252	
Difference: crude-weighted		1	0		1	0		-9	0		21	2		18	-5	

BC = breast cancer; SSA = sub-Saharan Africa; Difference is: ABC-DO – Globocan. Globocan 2018 estimates downloaded from https://gco.iarc.fr/ Reference: Bray F, Ferlay J, Soerjomataram I, Siegel RL, Torre LA, Jemal A. Global cancer statistics 2018: GLOBOCAN estimates of incidence and mortality worldwide for 36 cancers in 185 countries. *CA Cancer J Clin* 2018; **68**(6): 394-424.;

eFigure. Distribution of age at breast cancer death in each WHO region, noting the percentage of breast cancer deaths occurring < 50 years. Data: Globocan 2018. Source: Bray F, Ferlay J, Soerjomataram I, Siegel RL, Torre LA, Jemal A. Global cancer statistics 2018: GLOBOCAN estimates of incidence and mortality worldwide for 36 cancers in 185 countries. *CA Cancer J Clin* 2018; **68**(6): 394-424.

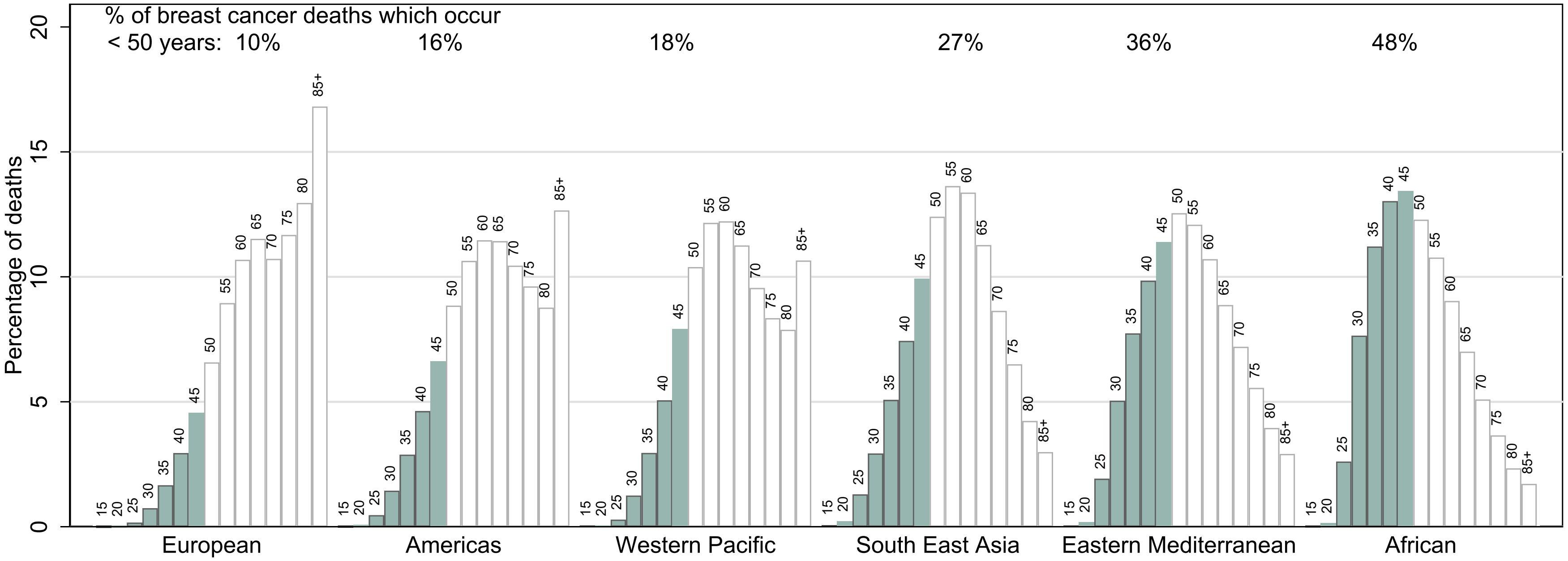

Legend: Each bar is labelled with the lower limit of a 5-year age-at-death band, or age 85+ years. Deaths under age 50 are shaded.
